# High-Stakes Decision-Making Before ECT: The Power of the Pause

**DOI:** 10.1192/bjo.2026.11798

**Published:** 2026-06-30

**Authors:** Chioma Ekwutosi Maduka

**Affiliations:** Leeds and York Partnership NHS Foundation Trust, Leeds, United Kingdom

## Abstract

**Aims:**

Background

Electroconvulsive Therapy (ECT) is among the most effective treatments for severe and life-threatening psychiatric illness, yet its use in medically complex patients demands refined clinical judgment.

With rising multimorbidity and increasing prevalence of implanted medical devices, clinicians are more frequently required to balance urgency against evolving physical risk.

This presentation introduces the concept of the *therapeutic pause;* not as delay, but as an active, patient-centered safety strategy, demonstrating how structured reflection and multidisciplinary collaboration can transform high-risk uncertainty into safe, timely intervention.

The aim is to highlight how deliberate pauses at critical decision points can improve both clinical outcomes and patient safety.

**Methods:**

Case Reports

Two urgent liaison psychiatry referrals for ECT are presented.

Case 1 involved a 72-year-old man with schizophrenia and mild learning disability, admitted with catatonia, severe self-neglect, and nutritional failure requiring nasogastric feeding. He had a proximal deep vein thrombosis (DVT) diagnosed three weeks earlier and hyperkalaemia, raising concern about embolic risk and physiological stress during ECT.

Case 2 involved a 62-year-old woman with psychotic depression and catatonia, who had an implanted spinal cord stimulator (SCS) for chronic pain, creating potential risks of electrical interference and device damage.

In both cases, ECT urgency was balanced against medical risk using targeted investigations, literature review, consultation with the ECT Accreditation Service (ECTAS) Knowledge Hub, and early multidisciplinary input.

**Results:**

In Case 1, repeat Doppler imaging confirmed resolution of the proximal DVT and electrolyte abnormalities were corrected, allowing ECT to commence three days later. By the third and fourth treatments, the patient showed striking recovery: spontaneous speech, independent mobility, recognition of staff, and restoration of oral intake, facilitating discharge after completing the course.

For Case 2, collaboration with pain specialists and device manufacturers enabled safe deactivation, monitored ECT delivery, and post-treatment device integrity checks, followed by clear improvement in psychomotor function, affect, and nutritional status.

These cases demonstrate that ECT risk is rarely absolute; it is dynamic and often modifiable. In both cases, pausing prevented both premature exposure to harm and unnecessary therapeutic delay.

**Conclusion:**

Pausing before ECT is not clinical indecision; it is clinical leadership. Individualized assessment, proactive multidisciplinary collaboration, and informed use of national guidance allow clinicians to navigate the interface between psychiatric urgency and physical vulnerability.

As ECT increasingly serves patients with complex comorbidity, reflective practice must replace routine automation. Thoughtful pauses protect patients, strengthen decision-making, and ultimately enable ECT to deliver its life-saving potential.

In complex ECT, the safest action may begin with a pause.

